# Comparative Analysis of Bioactive Compounds in Two Globe Artichoke Ecotypes Sanitized and Non-Sanitized from Viral Infections

**DOI:** 10.3390/plants12081600

**Published:** 2023-04-10

**Authors:** Roberta Spanò, Stefania Fortunato, Vito Linsalata, Isabella D’Antuono, Angela Cardinali, Maria Concetta de Pinto, Tiziana Mascia

**Affiliations:** 1 Department of Soil, Plant and Food Sciences, University of Bari “Aldo Moro”, Via Amendola 165/A, 70126 Bari, Italy; 2 Department of Bioscience, Biotechnology and Environment, University of Bari “Aldo Moro”, Via E. Orabona 4, 70124 Bari, Italy; 3 Institute of Science of Foods Production (ISPA), CNR Via Amendola 122/O, 70126 Bari, Italy

**Keywords:** artichoke ecotype, artichoke transcriptome, bioactive compounds, lignin, peroxidase, virus sanitation

## Abstract

Globe artichoke ecotypes sanitized from plant pathogen infections are characterized by high vegetative vigor, productivity, and quality of capitula. The recent availability on the market of these plants has renewed the interest of farmers and pharmaceutical industries in the crop. Globe artichoke exhibits interesting nutraceutical properties due to the high content of health-promoting bioactive compounds (BACs), such as polyphenols, that could be extracted from waste biomass. The production of BACs depends on several factors including the plant portion considered, the globe artichoke variety/ecotype, and the physiological status of the plants, linked to biotic and abiotic stresses. We investigated the influence of viral infections on polyphenol accumulation in two Apulian late-flowering ecotypes “Locale di Mola tardivo” and “Troianella”, comparing sanitized virus-free material (S) vs. naturally virus-infected (non-sanitized, NS) plants. Transcriptome analysis of the two ecotypes highlighted that differentially expressed genes (DEGs), in the two tested conditions, were mainly involved in primary metabolism and processing of genetic/environmental information. The up-regulation of the genes related to the biosynthesis of secondary metabolites and the analysis of peroxidase activity suggested that their modulation is influenced by the phytosanitary status of the plant and is ecotype-dependent. Conversely, the phytochemical analysis showed a remarkable decrease in polyphenols and lignin accumulation in S artichokes compared to NS plants. This unique study analyzes the potential of growing vigorous, sanitized plants, in order to have high amounts of ‘soft and clean’ biomass, finalized for BAC extraction for nutraceutical purposes. This, in turn, opens new perspectives for a circular economy of sanitized artichokes, in line with the current phytosanitary standards and sustainable development goals.

## 1. Introduction

The growing interest in biodiversity has made it possible to renew the competitiveness of several neglected plant species, varieties, and ecotypes worldwide. The Mediterranean basin boasts many species with interesting agronomic and qualitative traits that are attractive for farmers, nursery plants, processing industries, and markets for fresh consumption. Among these, globe artichoke (*Cynara cardunculus* L. var. *scolymus*) is gaining commercial interest for its nutritional and health-promoting benefits due to the high content of antioxidants, polyphenols, oligosaccharides, fibers, and minerals. These compounds, also referred to as bioactive compounds (BACs), have important pharmaceutical properties since they are hepatoprotective, anticarcinogenic, antioxidative, antibacterial, cholesterol-reducing, diuretic [1,2,3], and neuroprotective, the latter due to the recently recognized acetylcholinesterase inhibitory activity [4]. Artichoke production occurs in several regions of the Mediterranean area, and Apulia (southern Italy) is one of the major producers (I-Stat 2022, accessed on 15 September 2022, http://dati.istat.it/) hosting the most abundant in situ biodiversity of the *Cynara* spp. germplasm [5,6,7,8,9,10].

According to market demand, traditionally cultivated artichoke varieties are limited in number, grown for their large fleshy immature inflorescences (capitula), that are widely consumed in both fresh and preserved form. Globe artichoke varieties and ecotypes are defined on the basis of their capitula appearance and harvest time. Early flowering types can be induced to produce capitula between autumn and spring, whereas late flowering types produce capitula during spring and early summer. Such varietal traits are preserved via vegetative propagation, with limited selection criteria. Unfortunately, the criteria do not include the evaluation of the phytosanitary status of the offshoots collected in autumn–winter of the previous year or of the buds (ovules) collected in summer. As for most vegetatively propagated crops, the lack of proactive selection for mother plants with a good sanitary status has led over time to the accumulation of several pathogens in globe artichokes, such as vascular fungi (mainly *Verticillium* spp.) and viruses for which no efficient control measures are available [10,11]. These phytosanitary conditions lead to a significant loss of biodiversity and a decrease in crop quality and productivity. To prevent phytosanitary risks and the consequent decline in germplasm sources, a sanitation protocol based on the combination of in vitro meristem tip culture and thermotherapy should be applied for the production of virus-free standard material [10]. The reason for this double strategy is related to the distribution that different viruses may have in plant tissues. Some viruses infect epidermal cells, phloem, and parenchyma whereas others invade meristem tips from where they are transmitted to progeny through seeds [11]. Thus, different sanitation approaches might be necessary to clean plants from these systemic pathogens. On the other side, the application of sanitation protocols increases plant vigor and provides available procedures for the fast propagation of sanitized germplasm in nursery plants [10]. The rapid growth rate of sanitized virus-free plants (S), compared to naturally virus-infected artichokes (non-sanitized, NS), allows yield increase, which consists of a high number of capitula and a huge amount of leaf biomass (Supplementary Figure S1) useful for BAC or lignin extraction. In particular, lignin is an essential component of plant cell walls, providing the necessary structural support, but it may be used to produce renewable building blocks or, after depolymerization, converted into value-added products such as bioethanol and biobutanol [12,13,14]. The valorization of S artichoke brings positive effects not only on the food products, normally constituted by the ‘heart’ of the flower head and some inner bracts, but also on the 70–80% of the total plant part that is usually discarded [1,3,15,16] and that could be used in medicinally valuable phenolic-rich extract production.

In this scenario, the effects of sanitation on the physiology and/or morphology of artichoke plants and the production of BACs are largely unknown. Surface sterilization of plant material with a 20% (*v*/*v*) of a commercial bleach solution, meristem tip collection combined with at least two rounds of in vitro tissue culture in the presence of hormones, vitamins, and growth regulators followed by one or two rounds of thermotherapy [10] can lead to many changes that are not obviously all positive [17]. In particular, among BACs, the accumulation of mono- and di-caffeoylquinic acids (MCQ and DCQ, respectively) and flavonoids might be different between S and NS plants. Moreover, the sensitivity of genotypes to sanitation protocols proved to be highly variable [18]. Virus infections in NS plants are among the biotic stressors that might interfere with the quantity, purity, and efficacy of these BACs, which might not be the case in S ecotypes. Moreover, the different accumulation of precious BACs in different varieties and ecotypes positively influences their nutritional values and promotes their rediscovery and valorization [19,20]. The molecular and biochemical mechanisms regulating the production of specific bioactive metabolites in artichokes are still far from being clear. Few reports provide evidence concerning the role of gene families involved in the oxidative catabolism of phenolic compounds, such as peroxidases (POD) [15,21]. PODs are involved in enzymatic internal browning of plant tissues, changes in texture and flavor of globe artichoke capitula [22] and fruits [23], in lignification processes during plant growth [24,25], in plants wounded by atmospheric events, insects, snails, and rodents and in the defense against pathogens with the formation of phenolic polymers such as lignin and suberin [26]. The accumulation of peroxidase in response to pathogen inoculation has been demonstrated in several reports [27,28,29,30] but whether this enzyme accumulates also in response to sanitation and to which extent is unknown. The aim of this study was to understand the impact of a sanitation protocol on MCQ, DCQ, and flavonoid accumulation (henceforth referred to as BACs) and related biosynthetic pathways in two artichoke ecotypes. We analyzed differences in transcriptome profile, BAC accumulation, POD activity, and lignin production in Apulian late-flowering ecotypes “Locale di Mola tardivo” (LM) and “Troianella” (TR), comparing virus-free S germplasm with NS plants harboring natural virus infection. Most previous works on globe artichoke have focused on plant morphology, characterization and properties of chemical compounds, yield, and early loss of capitula [11]. Only a few reports have analyzed the variation of polyphenol composition fractions in different developmental stages of the plant and artichoke ecotypes [31] but, to the best of our knowledge, there are no reports on the transcriptome and biochemical changes deriving from the application of sanitation protocols.

## 2. Results

### 2.1. Evaluation of Morphological and Qualitative Traits

**Figure 1:**
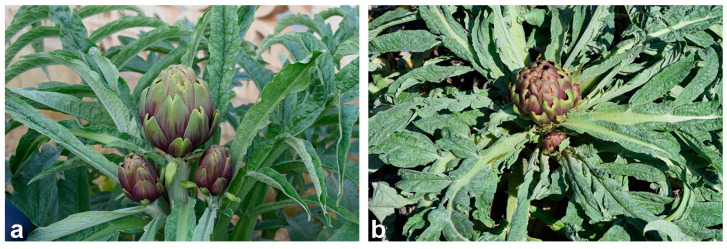
Artichoke biodiversity of late-flowering Apulian artichoke ecotypes (**a**) “Locale di Mola tardivo” and (**b**) “Troianella”.

Analysis of morphological and qualitative traits of LM and TR one-year-old plants showed clear differences related to the different genetic backgrounds (Figure 1), but also to the physiological responses of the two ecotypes exposed to variable open-field environmental conditions and agronomic treatments. 

On the contrary, plants obtained by in vitro meristem tip culture and thermotherapy revealed few differences between the two ecotypes considered for this study (Table 1).

In particular, sanitized LM and TR plants showed a reproductive cycle ranging from 8 to 10 months with the production in May of the oval central flower head and about 4 lateral heads on the main stem, followed by the differentiation of 3 to 4 lateral shoots. The plant height and leaf length in LM and TR ranged from 80 to 100 cm and 95–100 cm, respectively, with a plant diameter of 160 cm in LM and 190 cm in the TR ecotype. Although both showed similar lengths and diameters of the central flower heads, head weight was higher in TR than in LM.

### 2.2. Detection of Virus Infections

Samples of each of the LM and TR ecotypes were collected from a commercial field, transplanted and maintained in standardized conditions, and used as NS artichoke. Because virus distribution in globe artichoke may vary with the season and plant age, in addition to a generally symptomless infected plant, the success of any virus detection approach is strongly dependent on the choice of a proper time for sampling. Samples collected between early September and mid-November from young leaves of at least one-year-old plants give the best and most reproducible results [11]. NS plants did not show any viral symptoms at the time of sample collection, and thus occurrence of viral infections was assessed by dot blot analysis and then by RNA sequencing analysis (RNAseq). Preliminary screening of NS plant samples by polyprobe hybridization revealed the presence of at least one of the ten viruses covered by the polyprobe. Subsequently, dot blot hybridization of the same NS samples with virus-specific DNA probes showed the presence of artichoke latent virus (ArLV) and artichoke Italian latent virus (AILV) infection, whereas S plants proved to be virus-free.

**Figure 2:**
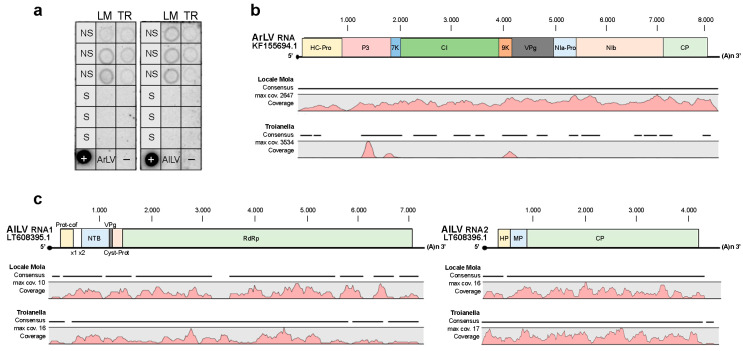
Virus detection in three biological replicates of non-sanitized (NS) and sanitized (S) plants of Locale di Mola (LM) and Troianella (TR) ecotypes. (**a**) Dot blot hybridization analysis using a specific digoxigenin-labeled DNA probe artichoke latent virus (ArLV) and artichoke Italian latent virus (AILV). Leaf extract of the healthy plant (−) and 1 ng of unlabeled target sequence (+) were used as controls. Hybridization signals were displayed using the Quantity One software (Bio-Rad Laboratories). Alignment and coverage analysis of RNA sequencing data obtained from LM and TR libraries of NS ecotypes against (**b**) ArLV RNA genome and (**c**) AILV RNA1 and RNA2 sequences. One of the three biological replicates for LM-NS and TR-NS was used to show read position alignment and coverage track results (pink color peaks) on virus genomes using the Integrative Genomics Viewer (IGV) tool.

Results from high-throughput sequencing (HTS) of three RNA preparations extracted from NS and S samples of the LM and TR ecotypes confirmed the absence of virus infection in S samples and the presence of ArLV and AILV infections in the NS, as already observed by dot blot hybridization (Figure 2 and Table S1).

RNAseq analysis of LM-NS and TR-NS libraries produced reads mapping to the full-length (8278nt) ArLV virus genome (Acc. N. KF155694.1) with a mean coverage fold of 989x and 217x for LM-NS and TR-NS, respectively (Figure 2b and Table S1). In detail, reads mapping distribution in LM-NS uniformly covered the entire virus genome, whereas in TR-NS reads mapped prevalently to P3 and 7K proteins and between the 9K and VPg gene regions. Reads mapping analysis was also performed against the full-length (11968nt) AILV virus genome divided between RNA-1 (Acc. N. LT608395.1) and RNA-2 (Acc. N.LT608396.1). The results showed a 4x mean coverage fold for both NS ecotypes (Figure 2c and Table S1) and an almost complete reads distribution over the RNA1 and RNA2 of the AILV genome.

Coverage analysis on virus genomes allowed the quantification of the titer of viruses detected by dot blot hybridization, accounting for 4.28 femtograms (fg) of reads mapped on the ArLV genome and 0.02 fg of reads on the AILV genome in LM-NS samples, whereas in TR-NS samples 0.94 fg of reads on ArLV genome and 0.03 fg of reads on AILV genome were detected (Table S1).

### 2.3. Comparative Analysis of Whole-Transcriptome of Artichoke Ecotypes

Sequencing of RNA samples on an Illumina platform produced an average of 29 million reads/libraries, with a high reading quality (mean quality score of 35.5 on a minimum reference value of 30) and a mean yield of 8.7 G bases. About 71% of reads were mapped against the *C. cardunculus* reference genome.

In order to validate the consistency of analyzed samples, we performed principal component analysis (PCA) which highlighted pronounced transcriptome changes in all tested samples after the sanitation process (Figure S2). In particular, Varimax rotation of the PCA scores plot in the sub-space factor 1 vs. factor 2 (accounting for 28.3% and 23.7% of the total variance, respectively) showed a clear separation between LM-NS and TR-NS, unlike the S samples of both ecotypes, which were closely related to each other.

The analysis of differentially expressed genes (DEGs) showed differences in each S sample compared with the NS of the same ecotype, as well as between LM-NS and TR-NS samples (Table 2).

DEGs in S samples compared with those in NS were 4269 for LM (Table 2 and Table S2) and 194 for TR (Table 2 and Table S3), respectively, with 103 genes in common (Table 2 and Table S4). The analysis of gene expression between the two NS samples revealed 4626 DEGs (Table 2 and Table S5) compared to only 75 genes in LM-S vs. TR-S (Table 2 and Table S6), with 56 genes in common between the two phytosanitary conditions (LM-NS vs. TR-NS compared to LM-S vs. TR-S; Table 2 and Table S7).

**Figure 3:**
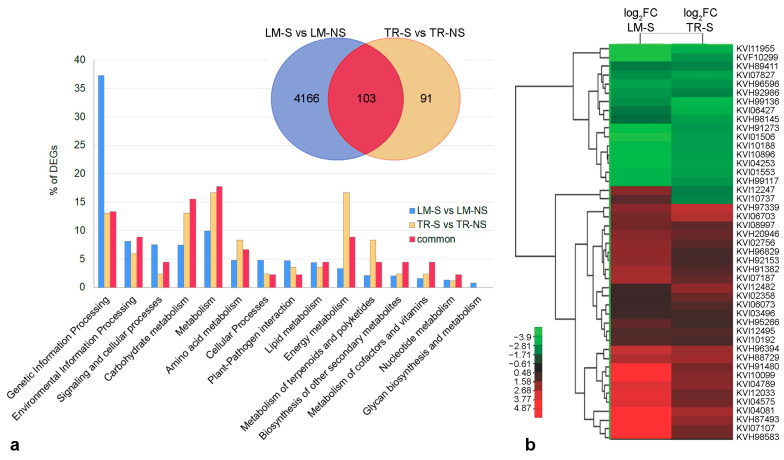
Functional classification and hierarchical clustering analysis of differentially expressed genes (DEGs) in sanitized (S) vs. non-sanitized (NS) samples of Locale di Mola (LM) and Troianella (TR). (**a**) The Venn diagram shows the number of DEGs (|log_2_FC| ≥ 1; FDR ≤ 0.05) in S vs. NS samples of LM and TR. The histogram shows the functional distribution of annotated DEGs exclusively modulated in response to the sanitation protocol (S vs. NS) in LM (4166 DEGs, blue bars), in TR (91 DEGs, yellow bars), and in common (103 DEGs, red bars) between LM-S and TR-S compared to NS samples. (**b**) The heatmap shows a hierarchical clustering analysis of expression levels (log_2_FC) of annotated DEGs (45 out of 103 genes) in common between LM-S and TR-S compared to NS samples.

KEGG analysis was used for the functional annotation of DEGs. The results from the comparison of S vs. NS samples showed that about 92.2% and 85.7% of DEGs in LM (Table S2) and TR (Supplementary Table S3), respectively, were involved in genetic and environmental information processing, carbohydrate, protein, lipid and energy metabolisms, and cellular processes (Figure 3a). Additionally, in LM, 7.8% (142 genes) of DEGs were involved in the biosynthesis of other secondary metabolites, whereas only 14.3% (12 genes) of the genes related to this biosynthetic pathway were differentially expressed in TR (Figure 3a). Considering the 103 DEGs in common between LM and TR, only 45 genes were annotated. These genes were mainly involved in the control of the cell’s primary metabolisms and in the regulation of genetic information (84.4%), while the remaining 15.6% (5 genes) were annotated on secondary metabolism with a similar expression profile between LM and TR (Figure 3b and Table S4).

**Figure 4:**
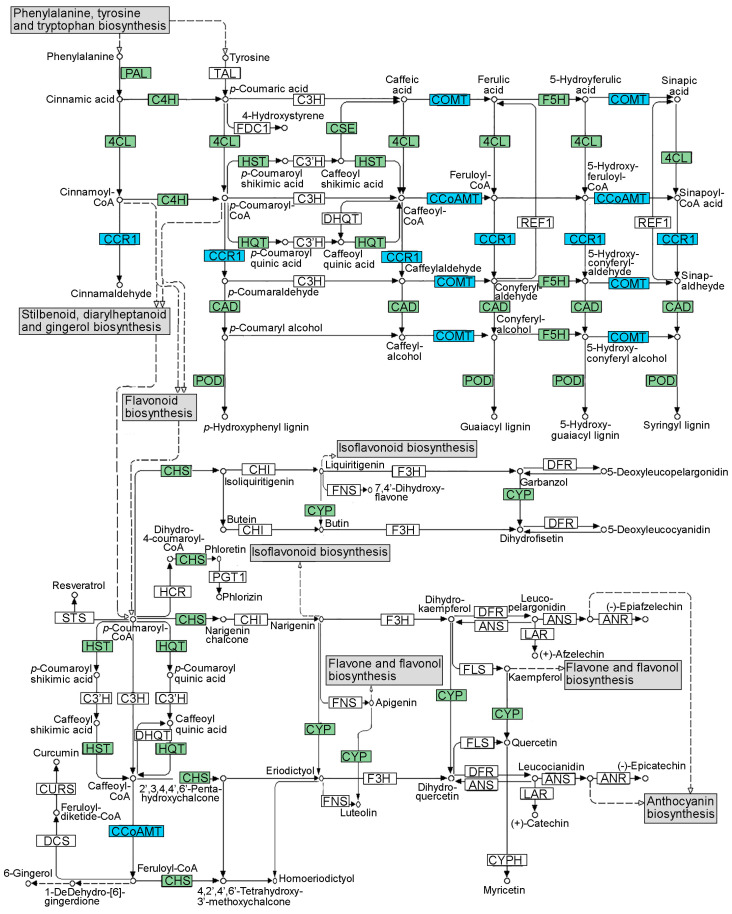
Schematic representation of secondary metabolite pathway map for phenylpropanoid, flavonoid, stilbenoid, diarylheptanoid, and gingerol biosynthesis. The positions in the map of the proteins encoded by the differentially expressed genes (DEGs) modulated in the LM and TR ecotypes, in the sanitized (S) vs. non-sanitized (NS) samples, are highlighted with blue boxes. The position of the proteins encoded by the DEGs observed only in the analysis between LM-S vs. LM-NS, is highlighted with green boxes. Protein abbreviations: 4CL, 4-coumaric acid:CoA ligase; ANS, anthocyanidin synthase; ANR, anthocyanidin reductase; C3H, *p*-coumaric acid 3-hydroxylase; C3′H, *p*-coumaroyl-shikimic acid/quinic acid 3-hydroxylase; C4H, cinnamic acid 4-hydroxylase; CAD, cinnamyl-alcohol dehydrogenase; CCoAOMT, caffeoyl-CoA *O*-methyltransferase; CCR1, cinnamoyl-CoA reductase 1; CHI, chalcone isomerase; CHS, chalcone synthase; COMT, caffeic acid 3-*O*-methyltransferase; CSE, caffeoyl shikimic acid esterase; CURS, curcumin synthase; CYP, flavonoid 3′-monooxygenase; CYPH, flavonoid 3′,5′-hydroxylase; DCS, phenylpropanoyl-diketide-CoA synthase; DFR, 5-deoxyleucopelargonidin:NADP+ 4-oxidoreductase; DHQT, caffeoyl-CoA:quinate *O*-(3,4-dihydroxycinnamoyl) transferase; F3H, flavanone,2-oxoglutarate:oxygen oxidoreductase; F5H, ferulic acid 5-hydroxylase; FDC1, ferulic acid decarboxylase; FLS, flavonol synthase; FNS, flavone synthase I; HCR, dihydro-4-coumaroyl-CoA:NADP+ 2,3-oxidoreductase; HST/HQT, hydroxycinnamoyl-CoA:shikimic/quinic acid hydroxycinnamoyltransferase; LAR, leucoanthocyanidin reductase; PAL, phenylalanine ammonia-lyase; PGT1, phlorizin synthase 1; POD, peroxidase; REF1, coniferyl-aldehyde dehydrogenase; STS, stilbene synthase; and TAL, tyrosine ammonia-lyase. The figure map was adapted from the KEGG database.

In detail, gene transcripts KVI08997 (caffeoyl-CoA *O*-methyltransferase, *CCoAMT*), KVI06073 (cinnamoyl-CoA reductase 1, *CCR1*), and KVI04575 (caffeic acid 3-*O*-methyltransferase, *COMT*), involved in the phenylpropanoid biosynthesis (Table 3 and Figure 4), were up-regulated in both ecotypes (Figure 3b). The *CCoAMT* gene is also involved in flavonoid, stilbenoid, diarylheptanoid, and gingerol biosynthesis. Moreover, gene transcripts KVI04081 and KVH87493, involved, respectively, in the biosynthesis of carotenoid and various plant secondary metabolite biosynthesis, were also up-regulated in both ecotypes (Figure 3b and Table 3). This set of 5 DEGs shared between LM-S and TR-S compared to NS plants (Figure 4, blue boxes) was considered for quantitative real-time PCR (qPCR) validation, together with the other 10 genes differentially expressed only in LM (Figure 4, green boxes).

Among the DEGs observed only in LM-S vs. LM-NS comparison, almost all genes showed significant up-regulation (FDR ≤ 0.05), except for the cinnamic acid 4-hydroxylase (*C4H*) and flavonoid 3′-monooxygenase (*CYP*) genes, accounting a log_2_FC of −1.8-fold and −1.1-fold, respectively (Table S2).

Several transcripts, annotated in the *C. cardunculus* genome, related to peroxidase proteins, showed significant up-regulation (FDR ≤ 0.05), with a total increase rate of 2.2-fold in the LM-S vs. LM-NS comparison. On the other hand, in TR-S samples, the same genes did not show any significant up-regulation (Table S8). Moreover, comparing samples of the two NS ecotypes, we observed a significant overexpression (1.5-fold) of *POD* genes in TR vs. LM, while transcript levels of peroxidase in S samples did not show any significant differences (Table S8).

**Figure 5:**
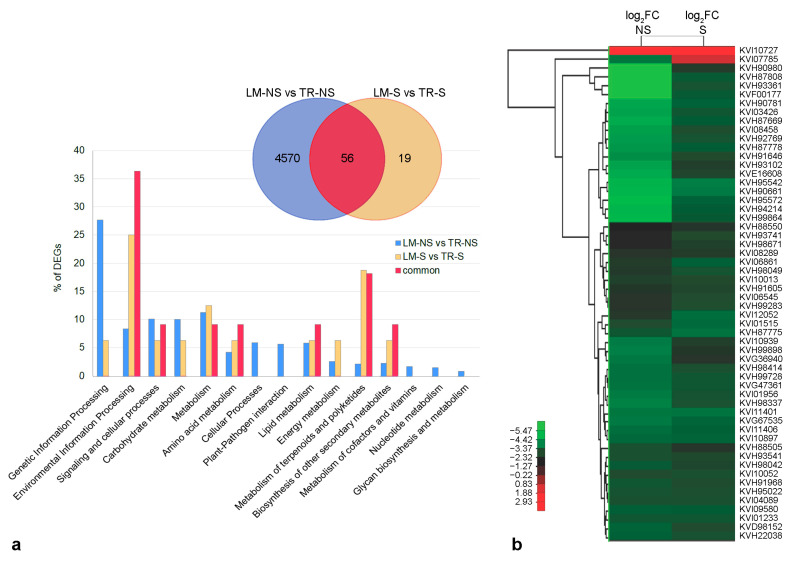
Functional classification and hierarchical clustering analysis of differentially expressed genes (DEGs) between Locale di Mola (LM) and Troianella (TR). (**a**) The Venn diagram shows the number of DEGs (FDR ≤ 0.05; |log_2_FC| ≥ 1) in LM compared to TR samples. The histogram shows the functional distribution of annotated DEGs exclusively modulated in LM compared to TR in non-sanitized (NS) condition (4570 DEGs, blue bars), in sanitized (S) condition (19 DEGs, yellow bars), and in common (56 DEGs, red bars) between NS and S samples. (**b**) The heatmap shows a hierarchical clustering analysis of expression levels (log_2_FC) of annotated DEGs in common between NS and S samples of the LM and TR ecotypes.

The analysis of the other DEGs between the two NS samples revealed transcriptome changes in genes mainly involved in genetic and environmental information processing, as well as in signaling and cellular processes and primary metabolisms (Table S5), while the comparison between S samples showed only 75 DEGs (Table S6), which were principally involved in environmental information processing, and primary, terpenoid, and polyketide metabolisms (Figure 5a). The transcriptome profiles of LM and TR after in vitro meristem tip culture and thermotherapy were comparable, and this similarity observed in S samples may be related to both growth in controlled conditions and virus-free status. Analysis of genes in common between the two phytosanitary conditions (LM-NS vs. TR-NS compared to LM-S vs. TR-S) showed 56 DEGs (Table S7), corresponding to the 75% of DEGs observed in the comparison between the LM-S and TR-S. These genes are involved in environmental information processing, and primary and secondary metabolisms (Figure 5a). Four DEGs, related to the transcript IDs KVH93541, KVH87775, KVH87778, and KVH88550 were also observed in the comparison between S and NS samples (Table S4) but were not annotated in the KEGG orthology. A preliminary analysis showed that genes related to transcripts KVH93541, KVH87775, and KVH87778 code the ankyrin repeat-containing protein mainly involved in salt stress tolerance through abscisic acid (ABA) signaling pathways, whereas the KVH88550 transcript was preliminary annotated as putative AB-hydrolase. All these genes were down-regulated in LM-NS and LM-S samples compared to TR-NS and TR-S, respectively. Heatmap analysis disclosed a marked difference in gene expression between the two NS ecotypes, with log_2_FC values ranging from −7.7 for KVH87808 (mannose-binding lectin) to 3.3 for KVI10727 (protein with unknown function), while gene expression level among S samples ranged from −3.2 for KVH95542 (glycoside hydrolase) to 2.5 for KVI10727 (Figure 5b, Table S7).

The DEGs *CCoAMT, CCR1, COMT,* and *NCED*, shared between the LM and TR ecotypes in the S vs. NS comparison, were validated by quantitative real-time PCR (qPCR) using the *Elongation factor 1 alpha* (*EF-1a*) of artichoke as housekeeping gene (HK). We also included the DEGs observed only in the LM-S vs. LM-NS comparison in the qPCR analysis: *4CL*, 4-coumaric acid:CoA ligase; *C4H*, cinnamic acid 4-hydroxylase; *CAD*, cinnamyl-alcohol dehydrogenase; *CHS*, chalcone synthase; *CSE*, caffeoyl shikimic acid esterase; *CYP*, flavonoid 3′-monooxygenase; *F5H*, ferulic acid 5-hydroxylase; *HST/HQT*, hydroxycinnamoyl-CoA:shikimic/quinic acid hydroxycinnamoyltransferase; and *PAL*, phenylalanine ammonia-lyase; *POD*, peroxidase. For this purpose, specific primers for the selected DEGs were synthesized (Table S9) to amplify cDNAs obtained from RNA preparations of NS and S samples in three biological replicates. PCR efficiency ranged from 92.2 to 112.1% with a regression coefficient (R^2^) of around 0.98.

**Figure 6:**
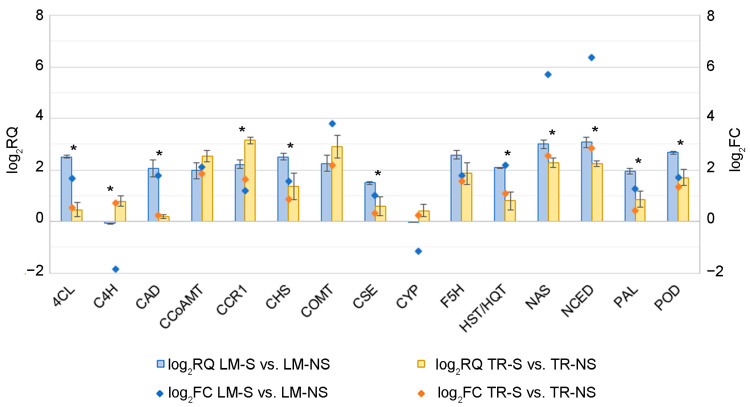
Validation of RNAseq data by quantitative real-time PCR (qPCR) of genes involved in the biosynthesis of secondary metabolites in sanitized (S) vs. non-sanitized (NS) plants of Locale di Mola (LM) and Troianella (TR). Columns represent log_2_ of gene expression value (RQ) obtained from qPCR calculated as the mean of three biological replicates. The NS condition was used as a reference plant for gene expression normalization, as a non-treated plant ecotype. Elongation factor 1 alpha (*EF-1a*) was used as a housekeeping gene for target gene normalization. For each gene, asterisks (*) indicate log_2_RQ values with statistically significant differences for *p* ≤ 0.05 performing a Tukey post hoc ANOVA test. Square points represent log_2_ of FC obtained from DESeq2 analysis. Gene abbreviations: *4CL*, 4-coumaric acid:CoA ligase; *C4H*, cinnamic acid 4-hydroxylase; *CAD*, cinnamyl-alcohol dehydrogenase; *CCoAOMT*, caffeoyl-CoA *O*-methyltransferase; *CCR1*, cinnamoyl-CoA reductase 1; *CHS*, chalcone synthase; *COMT*, caffeic acid 3-*O*-methyltransferase; *CSE*, caffeoyl shikimic acid esterase; *CYP*, flavonoid 3′-monooxygenase; *F5H*, ferulic acid 5-hydroxylase; *HST/HCT*, hydroxycinnamoyl-CoA:shikimic/quinic acid hydroxycinnamoyltransferase; *NAS*, Nicotianamine synthase; *NCED*, 9-cis-epoxycarotenoid dioxygenase; and *PAL*, phenylalanine ammonia-lyase; *POD*, peroxidase.

QPCR confirmed the up-regulation (log_2_RQ ≥ 1) of almost all DEGs of the secondary metabolite pathway in S plants compared to NS samples, except for *C4H* and *CYP* genes in LM-S samples, as already observed in transcriptome analysis (Figure 6). The genes *4CL*, *CAD*, *CHS*, *CSE*, *HST*/*HQT*, *NAS*, *NCED*, *PAL*, and *POD* were significantly more expressed in LM compared to TR samples, while *C4H* and *CCR1* were significantly more expressed in TR samples compared to LM.

In TR samples, the gene expression analysis by qPCR between S and NS samples showed the up-regulation of *CCoAMT*, *CCR1*, *COMT*, *NCED*, and *NAS* genes, as expected based on transcriptome analysis. Moreover, up-regulation was also observed for *CHS*, *F5H*, and *POD* genes, although the false discovery rate (FDR, according to the Benjamini–Hochberg test procedure) of log_2_FC was not significant. However, the unadjusted *p*-value was significant only for the *F5H* and *POD* genes (*p* ≤ 0.05, Table S10), as well as for the *HST/HQT* gene, slightly up-regulated in qPCR analysis.

### 2.4. BAC Chemical Analysis and Characterization

**Figure 7:**
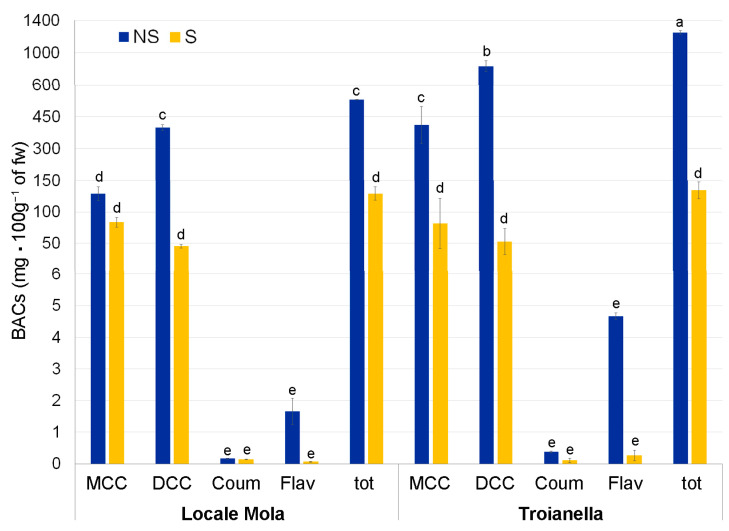
High-pressure liquid chromatography with diode array detection (HPLC-DAD) quantification of mono- (MCQ), and di- (DCQ) caffeoylquinic acids, coumaric derivates (Coum), and flavonoids (Flav) over total polyphenol (tot) content in samples of non-sanitized (NS) and sanitized (S) Locale di Mola and Troianella ecotypes. Letters indicate statistically significant differences for *p* ≤ 0.01 performing a Tukey post hoc ANOVA test.

Chemical analysis of BACs obtained from leaf extracts revealed a different accumulation of total polyphenols between S and NS samples. In samples of the LM-S ecotype compared to LM-NS, polyphenol content decreased by four-fold (Figure 7). The most abundant classes of polyphenols were MCQ and DCQ acids, with the DCQ significantly higher (almost four-fold) than MCQ in LM-NS. After the sanitation, their amounts became almost the same. Coumaric acid derivatives and flavonoids did not show significant changes attributable to the sanitation process. The same trend, but with strong differences between NS and S samples, was evident in TR with an increase in total polyphenols in TR-NS vs. TR-S of about 15-fold (Figure 7). In particular, they were significantly higher (about 15-fold) than in TR-S. In addition, for the TR ecotype, the MCQ and the DCQ were the most abundant polyphenol classes, with DCQ two times higher than MCQ. In TR-S samples, no statistically significant differences were observed between MCQ and DCQ after sanitation, although the total polyphenols identified were lower than in TR-NS samples. After sanitation, the coumaric acids derivatives did not show significant differences; similarly, flavonoids did not change between NS and S plants, although their decrease was more evident in TR-S compared to that observed for LM-S.

A detailed comparison of the BAC chromatograms of S and NS samples in the two ecotypes showed a similar profile of the identified and most accumulated phytochemical compounds in the two conditions (Figure S3) although about 1% more unknown peaks were recorded in the S than in NS samples (Figure S4).

In conclusion, after the sanitation protocol, the polyphenol content underwent an important reduction, which was substantially similar in the two sanitized ecotypes, notwithstanding some minor differences observed in the TR ecotype.

### 2.5. Peroxidase Activity and Lignin Content

**Figure 8:**
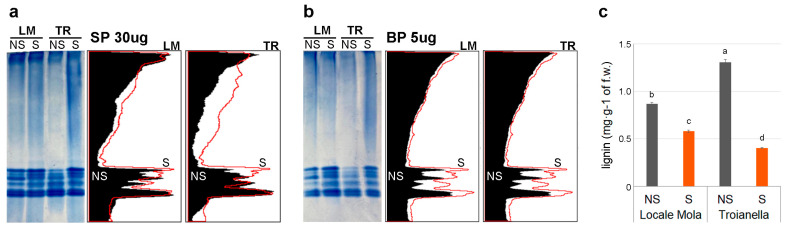
Analysis of artichoke peroxidase activity and lignin content accumulation in Locale di Mola (LM) and Troianella (TR) artichoke samples. Native-PAGE of (**a**) 30 μg of soluble (SP) fraction and (**b**) 5 μg of bound (BP) protein fraction of the LM and Troianella TR ecotypes. POD activity assay staining of gels was analyzed by ImageJ software to compare POD activity in non-sanitized (NS, black profile) and sanitized (S, red profile) plants. The histogram shows (**c**) lignin content in each condition tested. Letters indicate statistically significant differences for *p* ≤ 0.05 performing a Tukey post hoc ANOVA test.

To evaluate the response to oxidative stress of both ecotypes in the two phytosanitary conditions, the peroxidase activity in NS and S samples for TR and LM was determined by in-gel activity assay. Equal gel loading of total soluble and cell-wall-bound protein extracts were confirmed by Coomassie brilliant blue staining (Figure S5a,b). Interestingly, S samples exhibited higher levels of peroxidase activity compared to NS. The increase in POD activity was observed in both soluble peroxidase (SP) and cell-wall-bound peroxidase (BP) enzyme fractions (Figure 8a,b). A detailed analysis of enzyme activity resulting from the two extracts showed that BP activity was significantly higher than that of SP because only 5 μg of BP extract was necessary for the assay compared to the 30 μg used for SP.

Since PODs are involved in lignin biosynthesis, lignin content in all conditions was quantified by measuring the quantity of the polymer in leaf extracts using a calibration curve (Figure S5c). S samples showed a reduction in lignin accumulation compared to NS samples and the decrease was more evident in TR (3.2-fold) than in LM (1.5-fold) (Figure 8c). The accumulation of lignin in NS samples negatively correlated with the POD activity recorded in native-PAGE analysis and in comparative transcriptome analysis between S and NS samples, whereas it was more directly related to virus infection and BAC accumulation (Table S8).

## 3. Discussion

### 3.1. Ecotypes’ Adaptation to Environments and Phytosanitary Status

The Mediterranean basin, and Italy in particular, harbor the richest collection of globe artichoke cultivated germplasm and represent a reservoir of in situ biodiversity [32,33]. Clonal propagation and sexual reproduction in artichoke have played an important role in the domestication of the crop [34], but some ecotypes have remained limited to their geographic areas of origin and are often identified by vernacular names.

Besides the use of harvested capitula for human consumption, the entire plant represents a significant source of biopharmaceuticals [35,36,37], lignocellulosic biomass, and paper pulp [38,39]. The adaptation of ecotypes to different local environments has induced changes not only in the morphological traits of plants but also in the content of chemical compounds and in the quantity and properties of BACs [16,40]. The analysis of morphological traits of the two late-flowering artichoke ecotypes, LM and TR, traditionally grown in their area of origin, has shown differences in the overall behavior of the plant. 

Samples of plants of the two ecotypes collected from an open field were compared with samples of plants obtained by sanitation protocols and conserved ex situ in a plant nursery. To harmonize the experimental conditions, samples of both ecotypes were transplanted and grown in a dedicated greenhouse with controlled temperature, relative humidity, and light photoperiod. Although naturally infected plants are usually asymptomatic, dot blot hybridization analyses on leaves of artichoke plants collected from commercial crops grown in an open field revealed the presence of a mixed infection of ArLV, genus *Macluravirus*, family *Potyviridae* [41] and AILV, genus *Nepovirus*, family *Secoviridae* [42].

The presence of ArLV has a damaging effect on crop production mainly due to yield losses (around 50%) and delayed harvesting (Figure S1) [43]. The occurrence of the virus is associated with a significant decrease in the size and number of marketable heads, color breaking and premature opening of head scales, as well as shortening of the head stalk. Moreover, ArLV infection reduces the stress tolerance of plants leading to a low survival rate of plantlets (6.5%) subjected to heat treatments during in vitro meristem tip culture, compared to plants infected by AILV (90.9%) [44]. The incidence of ArLV infection is generally high since the virus is efficiently transmitted by aphids with a non-persistent modality, so the possibility of control is limited because vectors can transmit the virus before being killed by pesticide molecules. Additionally, while recurrent aphid infestations in a single vegetative season may cause, over time, the accumulation of ArLV in artichoke tissues, this is not the case for AILV. Compared to ArLV, the AILV incidence is expected to be lower since it is transmitted by nematodes, which proceed slowly in the soil and are not widespread in all cultivation areas of globe artichoke. In artichoke, AILV infection is mainly symptomless, although in some cases it can cause the appearance of yellowing and loss of symmetry of the leaves, while in other crops, traditionally grown in the same areas (e.g., chicory), the virus can cause very severe symptoms. In artichoke, as well as in other susceptible crops, AILV is present in meristem tips and therefore can be seed-transmitted, being widespread in the Mediterranean basin as a result [43,45,46], often in mixed infections with ArLV [11,41]. Coverage analysis in artichoke crops analyzed in this study confirmed the higher incidence of ArLV infection (4.28 fg in LM-NS and 0.94 fg in TR-NS), compared to AILV (a mean of 0.025 fg in the two NS ecotypes). Thus, the increase in AILV infections in recent reports [11] may be linked to the vegetative propagation of artichoke crops through shoots and buds, which is routinely adopted by farmers, rather than to nematode transmission. Thus, the use of virus-free propagation material maintained ex situ in plant nurseries, and provided to farmers and breeders for the new plantings, seems to be the only proactive and sustainable approach to progressively reduce the inoculum of these viruses in the field. Current EU Directives 93/61/CEE and 93/62/CEE, as modified/adapted by the new Plant Health Regulation (EU) 2016/2031 and (EU) 2017/625, enforce nursery production to be based on virus-free and true-to-type certified stocks [10,47,48]. Moreover, it is well known that sanitized plants show improved qualitative and quantitative traits in open-field culture [10], as also observed in this study. The uniformity of morphological traits of S plants associated with increased plant height, diameter, and leaf length (Table 1) leads to a boost in plant growth and biomass production (Figure S1), which can compensate for the higher cost of planting material [49].

### 3.2. Modulation of Artichoke Ecotype Transcriptome in Sanitized Plants

Transcriptome analysis revealed a distinct response of the two ecotypes to virus infection, showing the modulation of 4269 genes in LM and only 194 genes in TR (Table 2). A different transcriptome profile was also observed in LM-NS compared to TR-NS samples (Table 2). On the other hand, only a few genes were differentially expressed between the two S ecotypes (Table 2), as also observed in the PCA score plots (Figure S2) analysis, probably due to the same sanitation protocol followed by homogeneous acclimation and growing under controlled nursery conditions. Several genes in common between ‘LM-NS vs. TR-NS’ and ‘LM-S vs. TR-S’ are involved in environmental information processing, while no common genes involved in genetic information processing, carbohydrate, and energy metabolisms have been found. This suggests that, in spite of the application of sanitation procedures, the LM and TR ecotypes retained their original genetic background. It is well known that artichoke cultivars show marked variability in the accumulation of BACs, especially in the flower head [31], but no reports are available about the synthesis of these compounds in virus-free plants yielded by a sanitation process regardless of the selected ecotype. In this study, the homogeneous growing conditions and the availability of germplasm with a controlled phytosanitary status have allowed the analysis of the expression pattern of key genes involved in the synthesis of BACs in the two globe artichoke ecotypes. 

The up-regulation of *CCoAMT*, *CCR1*, and *COMT* observed in LM and TR genes showed the different response of S plants compared to the infected ones (Figure 6 and Table S10). In LM-S, the up-regulation of *4CL*, *CAD*, *CHS*, *CSE*, *HST*/*HQT*, *F5H*, *PAL*, and *POD*, and the down-regulation of *C4H* and *CYP* was also validated by qPCR. In TR, these genes were not significantly differentially overexpressed in the RNAseq analysis (Table S10); however, the results from qPCR showed an overall up-regulation in S plants compared to NS ones (Figure 6). These DEGs are involved in the biosynthetic pathway of phenylpropanoids, or cynarin derivative compounds, caffeoylquinic acid, and hydroxycinnamic acid, as observed by functional analysis (Figure 4). Synthesis of phenylpropanoids is controlled in part by the key enzyme PAL. The accumulation of PAL transcripts has been described in response to biotic and abiotic stimuli as well as to lignin deposition in non-stressed plants [50]. Previous works have reported that the silencing of key enzymes of lignin synthesis increases the accumulation of flavonoids, indicating competition for substrates between flavonoid and lignin synthesis pathways [50]. The up-regulation of the *PAL* gene is associated with the increased expression of the genes *C4H*, *4CL*, *CSE, HST/HQT, F5H, COMT*, *CCoAOMT*, *CCR1*, and *CAD* involved in phenolic and/or lignin biosynthesis. The link between the phenylpropanoid/lignin pathways was also reported in RNAi silencing experiments of the *C4H* gene in *Artemisia annua* plants (family *Asteraceae*) [51]. *C4H* is highly up-regulated during abiotic stresses [52] and the down-regulation of this gene, reported in studies, may be related to the controlled conditions of nursery-grown S plants. The 4CL enzyme plays an essential role in the biosynthesis of coumarin skeletons in the phenylpropanoid pathway during lignin formation and is regulated in response to biotic/abiotic stimuli, as well as for *CSE, HST/HQT, F5H, COMT*, *CCoAOMT*, *CCR1*, and *CAD.* The activation of the lignin pathway has been already reported in samples showing the up-regulation of *CCR1* and *CAD* genes, but high transcript levels have been also observed during nutrient depletion conditions [50]. In this study, the observed increased levels of these related gene transcripts might be more associated with lignin biosynthesis. Moreover, *4CL-*, *CH4-*, and *CCoAMT*-related enzymes are also involved in flavonoid, stilbenoid, diarylheptanoid, and gingerol biosynthesis from phenylpropanoid derivatives, highlighting the multifunction of some key genes in the biosynthesis of secondary compounds. The expression of these genes, together with the *CHS*, leads to the accumulation of precursors for flavonoid biosynthesis, such as narigenin chalcone. Naringenin and dihydrokampferol are further downstream hydroxylated in anthocyanins by the CYP enzyme. The down-regulation of the *CYP* gene observed in LM-S vs. LM-NS samples, as well as in TR with a similar trend, suggest the reduction of anthocyanin accumulation in favor of other secondary compounds and/or lignin biosynthesis. Overall, these observations confirm that plant response to diverse stimuli and the diversion of carbon flux in the pathways may be finely regulated through a common controlling mechanism. 

Furthermore, the *NCED* gene, involved in the biosynthesis pathway of carotenoids, which are known to have high antioxidant properties of biomedical and health interest, was up-regulated in S samples compared to NS. The *NCED* gene is also involved in the biosynthesis of abscisic acid that coordinates plant growth and development in response to environmental changes. The *NAS* gene, involved in the biosynthesis of other secondary metabolites, was also up-regulated. The related enzyme acts as a sensor of the physiological iron status with the production of nicotianamine, an iron chelator [53], playing an important role in the long-distance translocation of the iron from the cell wall of roots to the shoots, when necessary [54,55,56]. 

Overall, the qPCR results validated the RNAseq data and confirmed the gene modulation observed in S plants compared to NS samples of the same ecotype.

### 3.3. Virus-Free Status Decreases the Accumulation of Polyphenols

The comparison of data obtained from the transcriptome analysis with the data of leaf polyphenolic characterization has highlighted that the up-regulation of DEGs in S plants did not correspond to an increased accumulation of polyphenols, flavonoids, and coumaric derivatives. This discrepancy may be due to the involvement of the observed DEGs in multiple pathways in addition to that of secondary metabolite biosynthesis (Table 3 and Figure 6), as also reported in the KO entry card of each gene analyzed. Therefore, a positive and unique correlation of the overexpression of these genes should not be expected only with the higher accumulation of the secondary metabolites analyzed. Moreover, the sanitation protocol induces a strong change in plant fitness, such as an increase in plant height, diameter, and leaf length. Thus, it is plausible that the increase in plant vigor and biomass implies a different modulation of all metabolic pathways in S plants compared to NS ones.

Given that artichoke plants are a rich source of polyphenols, the chemical composition of artichoke extracts has been well characterized. Among polyphenols, MCQ and DCQ are the major phytochemicals where the chlorogenic acid (5-*O*-caffeoylquinic acid) is the most abundant (39%), followed by 1,5-*O*-dicaffeoylquinic acid (21%) and 3,4-*O*-dicaffeoylquinic acid (11%), while cynarin (1,3-*O*-dicaffeoylquinic) is present at a lower percentage (1.5%). The total content of caffeoylquinic acids in artichoke depends on the physiological status of the tissues, ranging from about 8% in young leaves to less than 1% in senescent tissues. These compounds are responsible for the appearance of browning reactions, which occur through enzymatic oxidation [57]. Other phenolic compounds extracted from artichoke samples belong to the flavonoid class, which includes the flavones apigenin (such as apigenin-7-*O*-glucoside and apigenin-7-*O*-rutinoside) and luteolin (such as luteolin-7-*O*-glucoside and luteolin-7-*O*-rutinoside) that, together with the coumaric derivatives (such as *p*-coumaric), contribute to the total antioxidant capacity of these extracts. Polyphenols are a group of plant metabolites that play an important role in plant defense by counteracting biotic and abiotic stresses [58], diminishing plant growth and yields. Plant defensive responses to biotic or abiotic stresses are costly in terms of energy needed [59,60]. The biosynthesis of polyphenols and other defense responses force plants to use their carbon nutrient molecules in the secondary metabolism, resulting in a reduction of growth rate in favor of the defense needs [1,50,60,61,62,63,64,65,66]. The analysis of BAC content revealed a higher accumulation of polyphenols in NS, resulting in virus infection, as confirmed by dot blot hybridization and RNAseq quantification (Figure 2). Conversely, S samples showed a more marked decrease in all specific BACs analyzed compared to their NS counterparts (Figure 7). Genes related to carbohydrate and energy metabolisms in the ‘S vs. NS’ comparison for both ecotypes showed significant differences (Figure 3), while no changes were observed in ‘LM-NS vs. TR-NS’ and in ‘LM-S vs. TR-S’ comparisons (Figure 5). Thus, the increased level of phenolic compounds seems to be associated with viral infections, whereas, as also reported in other studies [1,60], the virus-free condition enhances the growth rate and the yield of plant biomass. 

### 3.4. Correlation of High POD Activity with the Accumulation of Developmental Lignin in Sanitized Plants

In this scenario, the reinforcement of plant cell wall involves the production of cell-wall-bound ferulic acid, cinnamaldehydes, and sinapic acid polymers from caffeoyl-CoA, cinnamoyl-CoA, and caffeic acid, respectively (Figure 4). The resulting products may subsequently be converted to the corresponding alcohols that are incorporated into lignin (Figure 4). Therefore, the phenylpropanoid biosynthesis pathway was also evaluated by the analysis of lignin accumulation. The results indicate a higher cell wall lignification of NS plants compared to S ones, which might reflect the continuous exposure to abiotic and biotic stress in the open field, while S plants, grown in controlled conditions, are characterized by an increased vigor and youthfulness. Several studies report a difference in the chemical composition of lignin produced in response to stresses, also denoted as ‘defense lignin’, and the polymer produced during physiological plant growth called ‘developmental lignin’ [67]. Although the enzymatic pathway for the biosynthesis of the two types of lignin is similar, the use of phenolic metabolites depends on different signal transduction based on plant needs. In *Arabidopsis thaliana,* two isoforms of the *CCR* gene have been characterized, *AtCCR1* (Acc. N. AF320624.1), expressed during development, and *AtCCR2* (Acc. N. AF320623.1), participating in stress responses, thus involved in the synthesis of ‘defense lignin’ [68]. In this study, the up-regulation of the *CCR1* gene (Ccrd_015577, transcript ID KVI06073, Figure 6) might be attributed to the production of constitutive lignin. The gene shows a slightly higher percentage of identity (73% and 2 gaps) with *AtCCR1* and other *CCR1* genes in the NCBI database, compared to *AtCCR2* (72% of identity and 10 gaps). Transcriptome analysis of NS plants showed significant overexpression of the *Ccrd_01390*5 gene, coding the *CCR2* protein (transcript ID KVI07737), with a log_2_FC of 1.84 (FDR ≤ 0.05, Table S5) in TR-NS compared to LM-NS, while no significant difference was found between S plants (Table S6). This result could be related to the higher accumulation of ‘defense lignin’ in TR-NS where a higher accumulation of polyphenols was recorded (Figure 8c). Therefore, the higher accumulation of polyphenols and ‘defense lignin’ in NS plants, and especially in TR-NS, might be related to the biosynthesis of molecules whose accumulation is linked to the resistance process, while the activation of the same pathway in S artichokes might be associated with the improved physiological growth of healthy plants. The reduced amount of lignin and the herbaceous traits of leaves in S plants could facilitate BAC extraction from “less woody” leaf tissue and S plants can be considered as a more ‘soft’ biomass in a scale-up BAC extraction model. In a recent study, the application of biostimulants and nitrogen fertilization to different artichoke cultivars has been proposed as a strategy to favor the accumulation of polyphenolic compounds [31]. Therefore, in artichoke, the combinations of a sanitation protocol and agronomic practices could represent a promising strategy for maximizing the recovery of a high quantity of BACs for nutraceutical purposes.

In artichoke plants, PODs influence the phenolic content. Transcriptome and qPCR analysis revealed an up-regulation of *POD* genes in LM-S vs. LM-NS, as well as in TR-NS vs. LM-NS, whereas no significantly different expressions were observed in TR-S vs. TR-NS plants and in the comparison of the two S ecotypes (Figure 6 and Table S8). The different modulation of *POD* genes in NS plants seems to be ecotype-dependent and related to the physiological status of the plant in the open field, whereas the S condition reduces the difference in transcript levels between the two ecotypes. To be sure, plants use many defense strategies that could be associated with polyphenol accumulation and ‘defense lignin’ production to counteract pathogen infection. PODs are enzymes implicated in several physiological processes and are usually present in plants in a high number of isoenzymes, based on tissue developmental state, physiological status, and environmental factors. Their activity, in turn, also contributes to the development of internal browning in the heads [22,69,70]. Considering the last steps of phenylpropanoid biosynthesis related to lignin polymerization, the oxidation of monolignols (*p*-coumaryl, coniferyl, and sinapyl alcohols) is catalyzed by peroxidases. The evaluation of POD activity between the NS and S samples by native-PAGE showed a slight increase in enzyme activity in the latter. The higher POD activity of extracts in S plants was not directly correlated with the lignin accumulation and BAC production observed in NS samples in response to virus infection. The application of sanitation protocols, with the excision of plant meristem tip, resulted in stress like that caused by herbivorous insects and the subsequent production of reactive oxygen species (ROS). Polyphenols produced by plants react with ROS leading to the browning of explants and meristem damage [71,72]. In this study, the increase in POD activity observed in S samples could be related to a higher ability of sanitized plants, acquired during previous stressful events, to respond to oxidative stress occurring under biotic and abiotic stresses. This suggests that sanitized plants might also grow better in adverse environmental conditions, although the relationship between stress response and POD modulation deserves further investigation.

## 4. Materials and Methods

### 4.1. Plant Materials and Assessment of the Sanitary Status

In late September 2021, before the start of the artichoke production cycle, ten young offshoots 10–15 cm in length for each of the two Apulian late-flowering artichoke ecotypes, LM and TR, were collected with systematic random sampling [73] from commercial artichoke crops grown in the open field. LM is a clone of the better-known early-flowering Locale di Mola ecotype, mainly cultivated in its area of origin near Bari (Mola di Bari, Apulia, southern Italy), while TR comes from the area of Foggia (Troia, in the north of the Apulia Region). Selected plants did not show evident symptoms of ongoing abiotic or biotic stresses. Collected samples were transplanted into 18 cm diameter pots to be maintained ex situ in a greenhouse at 18–20 °C, 55–60% relative humidity (RH), and 16 h light/8 h dark photoperiod. These plants constituted the NS stocks for LM and TR. Another group of ten young offshoots 10–15 cm in length for each of the two ecotypes, LM and TR, were collected with systematic random sampling from primary source sanitized germplasm [10] grown ex situ in large pots of a commercial nursery (Vivaio F.lli Corrado, Torre Santa Susanna, Brindisi, Apulia, southern Italy), in an aphid-proof dedicated greenhouse. Collected samples were transplanted into 18 cm diameter pots to be maintained ex situ in a greenhouse at 18–20 °C, 55–60% RH, and 16 h light/8 h dark photoperiod. These plants constituted the S stocks for LM and TR. 

UPOV descriptors (International Union for the Protection of New Varieties of Plants, accessed on 15 September 2022, https://www.upov.int/test_guidelines/en/list.jsp) take into account agronomic and qualitative traits of the LM and TR artichoke ecotypes. In this study, UPOV descriptors were used to identify appropriate characteristics for the evaluation of plant distinctness, uniformity, and stability (DUS). Among them, particular attention was given to the total number of flower heads produced, height and average diameter of the plant in the open field, main stem diameter, leaf length [74], and market demand of each ecotype. For the UPOV characterization, ten NS and S plants for each of the two ecotypes were grown in a comparative field together with other NS and S artichoke varieties and ecotypes. The characterization was performed on one-year-old plants (Figure S1).

NS and S plant samples in triplicate for each of the two ecotypes were tested for the presence of the most commonly occurring and economically relevant viruses in globe artichoke, according to the Italian Ministerial Decree n.18-02/02/2021 and n.40-01/10/2022. The presence of artichoke Italian latent virus (AILV), artichoke latent virus (ArLV), artichoke mottled crinkle virus (AMCV), turnip mosaic virus (TuMV), tomato infectious chlorosis virus (TICV), bean yellow mosaic virus (BYMV), cucumber mosaic virus (CMV), pelargonium zonate spot virus (PZSV), tomato spotted wilt virus (TSWV), and tobacco mosaic virus (TMV) was preliminarily tested with a digoxigenin-labeled polyprobe, as described by Minutillo et al. [75]. Positive reactions to polyprobe hybridization were identified through hybridization with single probes specific for each virus covered by the polyprobe, according to Spanò et al. [10].

### 4.2. Total RNA Extraction, cDNA Preparation, and High-Throughput mRNA Sequencing

Total RNA was extracted from three biological replicates of NS and S samples for each of the LM and TR ecotypes, grinding 100 mg of leaf material in liquid nitrogen and EuroGOLD RNAPure^TM^ (EuroClone, Pero Italy) following the manufacturer’s instructions. RNA concentration was estimated using a Qubit RNA HS assay kit (ThermoFisher Scientific, Waltham, MA, USA), whereas agarose gel electrophoresis and the Bioanalyzer RNA 6000 Pico Labchip (Agilent Technologies, Santa Clara, CA, USA) were used to estimate RNA integrity and quality. Samples with RNA integrity number (RIN) ≥ 7 were rRNA-depleted and used to prepare complementary DNA libraries for sequencing on an Illumina HiSeq 2 × 150 bp reads platform (Azenta-GENEWIZ).

### 4.3. Mapping of Sequence Reads

Raw reads were pre-processed by quality filtering prior to expression analysis with the FastQC tool (www.bioinformatics.babraham.ac.uk/projects/fastqc/) on the Galaxy platform (https://usegalaxy.eu). Reads were aligned against the *Cynara cardunculus* genome sequence (Acc. N. GCA_001531365.1) using the RNA STAR alignment program [76]. Mapped reads were counted with FeatureCounts [77] and fragments per million mapped reads per kilobase exon (FPKM) were calculated. Differentially expressed genes (DEGs) were identified using the DESeq2 tool [78] with default parameters. Genes whose expression values obtained from the logarithm (to basis 2) of fold change (FC) among conditions tested was |log_2_FC| ≥ 1 and with a false discovery rate (FDR) ≤ 0.05 [79] were used for gene ontology functional enrichment analysis by using the KOALA annotation tool [80]. For the purpose of this study, it was assumed for a specific gene that a |log_2_FC| ≥ 1 for the condition treated vs. untreated means that sanitation induces a multiplicative change in the observed gene expression level of 2^−1^ = 0.5 compared to the non-sanitized condition, and the reported log_2_FC is per unit of change of that variable. Hierarchical clustering (HCL) analysis of DEG values in common among the conditions tested was based on Euclidian distance metric with average linkage agglomeration, and data matrix distribution was represented in a heatmap with colors ranging from green (down-regulated genes) to red (up-regulated genes).

In addition to molecular hybridization, virus presence and accumulation in each biological replicate were estimated by mapping reads unaligned to the *C. cardunculus* genome against ArLV (GeneBank Acc. N. KF155694.1) and AILV (GeneBank Acc. N. LT608395.1 and LT608396.1 for RNA1 and RNA2, respectively) genomic sequences by running the Bowtie2 alignment tool [81,82]. Reads alignment was visualized using Integrative Genomics Viewer (IGV) [83,84].

### 4.4. Validation of the RNA-Sequencing Results by Quantitative Real-Time PCR

Total RNA (1 μg) extracted from three biological replicates of NS and S samples for the LM and TR ecotypes was treated with a TURBO DNA-free kit (ThermoFisher Scientific) to remove DNA contaminants and primed with random hexamers for first-strand cDNA synthesis, with the Tetro cDNA synthesis kit (Bioline) according to the manufacturer’s instructions. The comparative cycle threshold (2^−ΔΔCt^) method corrected for PCR efficiencies was used to estimate the relative abundance of genes involved in the biosynthesis of secondary metabolites. Genes were selected on the basis of their |log_2_FC| ≥ 1 (FDR ≤ 0.05) obtained from DESeq2 analysis of S artichokes compared to NS plants and used to prepare primer pairs (Table S9): 4-coumaric acid:CoA ligase (*4CL*); cinnamic acid 4-hydroxylase (*C4H*); cinnamyl-alcohol dehydrogenase (*CAD*); caffeoyl-CoA O-methyltransferase (*CCoAOMT*); cinnamoyl-CoA reductase 1-like (*CCR1*); chalcone synthase (*CHS*); caffeic acid O-methyltransferase (*COMT*); caffeoyl shikimic acid esterase (*CSE*); flavonoid 3′-monooxygenase (*CYP*); ferulic acid 5-hydroxylase (*F5H*); hydroxycinnamoyl-CoA:shikimic/quinic acid hydroxycinnamoyltransferase (*HST/HQT*); nicotianamine synthase (*NAS*); 9-cis-epoxycarotenoid dioxygenase (*NCED*); phenylalanine ammonia-lyase (*PAL*); and peroxidase (*POD*). Elongation factor 1 alpha (*EF-1a*) was used as a housekeeping gene for target gene normalization [85]. Conditions for quantitative real-time PCR (qPCR) were those described previously [86,87,88]. Briefly, qPCR was set up in three technical replicates of 10 μL of 1X PowerUp Sybr Green Master Mix (Applied Biosystems), containing 15 ng of first-strand cDNA template, and 200 nM each of the forward and reverse primer pairs for each condition, according to the manufacturer’s instructions. Reactions were performed using a StepOne Real-Time PCR system (Applied Biosystems, Waltham, MA, USA) apparatus followed by melting curve analysis to determine the specificity of the reaction. PCR efficiency for each amplified fragment was derived from the slope of the regression line obtained by interpolating values from triplicates of five serial 1:2 dilutions of input cDNA amount and the relative Ct values using the StepOne software (Applied Biosystems).

### 4.5. Artichoke Polyphenol Extraction and HPLC Analysis

Ten g of fresh artichoke leaves collected from three NS and S samples for each of the two LM and TR ecotypes were freeze-dried and used for polyphenol extraction by refluxing with 100 mL of methanol/water (80:20, *v*/*v*), for 1 h at 100 °C. The extracts were filtered through a Whatman 1 paper, pooled, filtered at 0.45 µm, and stored at −20 °C until analysis, following the protocol of D’Antuono et al. [89]. High-pressure liquid chromatography with diode array detection (HPLC-DAD) analysis was performed using an Agilent 1260 Infinity system, equipped with a 1260 binary pump, 1260 HiP degasser, 1260 TCC thermostat, 1260 diode array detector, and Agilent Open Lab Chem Station Rev C.01.05 (35) software. The UV–visible absorption chromatogram was detected at 280 nm, 325 nm, and 360 nm. The separation was performed on a 4.6 × 250 mm reversed-phase Luna C-18 (5 μm) column (Phenomenex Torrance, California, USA), by gradient elution using methanol (eluent A) and water/acetic acid 95:5 (eluent B), according to D’Antuono et al. [89]. The gradient profile was: 85–60% B (0–25 min), 60% B (25–30 min), 60–37% B (30–45 min), 37% B (45–47 min), and 37–0% B (47–52 min). The flow rate was 1 mL/min, and the injection volume was 25 μL. The phenolic compounds were identified and quantified by the retention time, spectra, and response factors of the pure standards supplied by PhytoLab GmbH & Co. KG (Dutendorfer Str. 5-7, 91487 Vestenbergsgreuth Germany). In particular, for this study, the standards used for the identification of MCQ were: 1-*O*-Caffeoylquinic acid, 3-*O*-Caffeoylquinic acid, and chlorogenic acid. For the identification of DCQ, the standards were: cynarin (1,3-*O*-Dicaffeoylquinic acid), 1,4-*O*-Dicaffeoylquinic acid, 4,5-*O*-Dicaffeoylquinic acid, 3,5-*O*-Dicaffeoylquinic acid, 1,5-*O*-Dicaffeoylquinic acid, and 3,4-*O*-Dicaffeoylquinic acid. Finally, Apigenin-7-*O*-glucoside was used for flavonoid identification.

The quantification, expressed as µg/mL, was made by the calibration curves of the respective standards, except for 1-*O*-caffeoylquinic quantified as chlorogenic-acid-equivalent, 1,4-*O*-dicaffeoylquinic acid quantified as 1,5-*O*-dicaffeoylquinic-acid-equivalent, and the coumaric derivatives quantified as coumaric-acid-equivalent.

### 4.6. Determination of Artichoke Peroxidase Activity and Lignin Content

Peroxidase enzyme extraction was performed according to Survilla et al. [90] with minor modifications, as mentioned below. Artichoke leaves (350 mg) collected from three NS and S samples for each of the two LM and TR ecotypes were ground in liquid nitrogen and the resulting powder was suspended in 2 mL of 100 mM Tris-HCl buffer pH 7.5 containing 250 mM sucrose and protease inhibitors (0.1 mM PMSF). The suspension was vortexed for 2 min and centrifuged at 17,000× *g* for 30 min at 4 °C. The supernatant was recovered and used for soluble fraction (SP) enzyme activity assays. Pellets were resuspended in 1 mL of a salt solution (100 mM Tris-HCl pH 7.5 1 M NaCl, 1 mM CaCl_2_, and 1 mM MgCl_2_), and bound proteins (BP) from cell walls were extracted by vortexing for 30 min at 4 °C and centrifugation at 17,000× *g* for 5 min at 4 °C. The supernatant was recovered and dialyzed overnight at 4 °C against 100 mM Tris-HCl buffer pH 7.5 to remove salts prior to peroxidase activity assay. The extracts of the two POD forms were kept at –20 °C until analysis. The total protein in the crude extract was estimated by Bradford assay using a Bio-Rad protein assay dye reagent (Bio-Rad Laboratories) following the manufacturer’s instructions. Crude protein extracts consisting of 30 μg of SP fraction or 5 μg of BP fraction were separated on a vertical gel apparatus (Bio-Rad Laboratories) following the manufacturer’s instructions. Native-PAGE was carried out using stacking and separating polyacrylamide gels as described by Laemmli [91], with some modifications. After the electrophoretic run, the gel was incubated at room temperature (RT) for 5 min in 100 mM Tris-acetate buffer pH 5 containing 1 mM metossinaftol and 0.15 mM of H_2_O_2_. The colorimetric reaction was visualized after washing with distilled water. The blue color produced by POD activity was evaluated by using ImageJ software (Rasband, 1997–2018. https://imagej.nih.gov/ij/, 1997–2018).

Lignin content in artichoke samples was determined on a spectrophotometer by measuring the UV absorption at 280 nm of thioglycolic lignin in 0.5M NaOH produced in each extract. Briefly, 1 g of lyophilized leaf material was ground in 1:3 *w*/*v* of 99% ethanol, and, once homogenized, was centrifuged for 15 min at 14,000 rpm at RT. Pellets were washed with 99% ethanol prior to drying them at 60 °C for 24 h. The alcohol-insoluble residue (AIR) formed was weighed and 10 mg were resuspended in 1.75 mL of 98% thioglycolic acid and 2N HCl (1:6). Samples were incubated 4 h at 100 °C and then centrifuged for 15 min at 12,000 rpm. Pellets were washed with distilled water and resuspended in 0.5 M NaOH prior to incubating for 18 h at 25 °C with shaking. Extracts were then centrifuged for 20 min at 12,000 rpm and supernatants were collected, acidified with 300 μL of 36% HCl, and incubated for 4 h at 4 °C. After centrifugation of 20 min at 12,000 rpm 4 °C, pellets were resuspended in 0.5 M NaOH prior to measuring the thioglycolic lignin collected. Lignin content in each sample was derived from the calibration line obtained by interpolating values of five serial 1:2 dilutions in triplicates of 10 mg of pure alkali lignin (Sigma-Aldrich, St. Louis, MO, USA).

### 4.7. Statistical Analysis

Statistically significant differences for *p* ≤ 0.05 were assessed by one-way analysis of variance (ANOVA) with Tukey’s post hoc test, using Statistica software, version 7.0 (Stat Soft, Inc. 1984–2004, Tulsa, USA).

Principal component analysis (PCA) of the consistency of sample libraries was assessed by multivariate data analysis with the chemometrics agile tool (CAT) software, version 3.1.2 (http://www.gruppochemiometria.it/index.php/software).

## 5. Conclusions

Italy represents a center of diversity for the globe artichoke and their morphological, molecular, chemical, and physiological differences make some of them particularly suitable for the extraction of chemical compounds with interesting nutraceutical properties, such as antioxidant, anti-inflammatory, and neuroprotective properties. Moreover, the application of a sanitation protocol allows the production of virus-free propagation plants of particular interest for the nursery trade due to the increased rate of plant growth, as well of capitula production, but also for the preservation of valuable genetic resources and for the extraction of healthy promoting BACs, such as polyphenols. Virus species, their colonization, and distribution in plant tissues, as well as the choice of the sanitation protocol to apply, should be carefully evaluated for each plant genotype considered. On the whole, these could modify the response of the plant to virus eradication and lead to the accumulation of different amounts of metabolites. After the sanitation protocol, plants increase their photosynthetic rate and vitality, which is the basis of plant growth and development. The possibility of soilless cultivation of S plants in controlled conditions encourages their use as a source of BAC extraction for pharmaceutical purposes.

This work also aims to support the production of certified material in line with the current phytosanitary standards enforced at the European and national level. Moreover, the results contribute to achieving the current sustainable development goals, opening new perspectives on the growth of a circular economy for sanitized artichokes.

To our knowledge, this is the first RNAseq study in two Apulian late-flowering artichoke ecotypes “Locale di Mola tardivo” and “Troianella”, showing that the application of a sanitation protocol and the virus-free condition of the resulting germplasm modulated the different expression of several genes and polyphenol composition, which appear to be the most significant novelties of the results.

## Figures and Tables

**Table 1:** Morphological and qualitative traits of Locale di Mola and Troianella artichoke ecotypes.

UPOV n. ^1^	Feature	Artichoke Ecotypes’ Feature Expression ^2^	
		**Locale di Mola**	**Troianella**
	Transplant	July–September	July–September
28	Central flower head production	December–May	February–May
	Reproductive cycle	8–10 months	8–10 months
1	Plant height	100 cm	80 cm
2	Number of lateral heads on the main stem	4	4
5	Main stem diameter	2 cm	2.5 cm
8	Leaf length	95 cm	100 cm
	Plant diameter	160 cm	190 cm *
23	Central flower head length	11.5 cm	10.5 cm
24	Central flower head diameter	8 cm	9 cm
25	Central flower head weight	180 g	220 g *
26	Central flower head shape	oval	oval
51	Shoots production	3–4	3–4

^1^ UPOV descriptors used for the morphological characterization of artichoke varieties. ^2^ Feature expression represents the average of ten one-year-old plants of the same ecotype; * indicates a statistically significant difference between the two ecotypes for *p* ≤ 0.05.

**Table 2:** The number of differentially expressed genes (DEGs) in sanitized (S) and non-sanitized (NS) artichoke samples of Locale di Mola (LM) and Troianella (TR).

Comparison Factors	n. of DEGs ^1^	% of DEGs ^2^	n. of DEGs in Common ^3^
LM-S vs. LM-NS	4269	16.10	103
TR-S vs. TR-NS	194	0.73	
LM-NS vs. TR-NS	4626	17.45	56
LM-S vs. TR-S	75	0.28	

^1^ DEGs with an expression value of |log_2_FC| ≥ 1 for *p* ≤ 0.05 adjusted for multiple testing with the Benjamini–Hochberg procedure which controls false discovery rate (FDR). ^2^ Percentage of DEGs out of the 26,505 annotated genes of the *C. cardunculus* genome. ^3^ DEGs in common were obtained by ‘LM-S vs. LM-NS’ compared to ‘TR-S vs. TR-NS’, and ‘LM-NS vs. TR-NS’ compared to ‘LM-S vs. TR-S’.

**Table 3:** Differentially expressed genes (DEGs) involved in the biosynthesis of secondary metabolites in common between sanitized Locale di Mola (LM) and Troianella (TR). The relative protein description, KEGG orthology (KO) entry, and EC number are also indicated in the table.

Gene Name	Gene ID	Transcript ID	KO	Protein Description	EC
				**Phenylpropanoid biosynthesis**	
*CCoAMT*	Ccrd_012618	KVI08997	K00588	caffeoyl-CoA *O*-methyltransferase	2.1.1.104
*CCR1*	Ccrd_015577	KVI06073	K09753	cinnamoyl-CoA reductase 1	1.2.1.44
*COMT*	Ccrd_017109	KVI04575	K13066	caffeic acid 3-O-methyltransferase	2.1.1.68
				**Flavonoid biosynthesis**	
*CCoAMT*	Ccrd_012618	KVI08997	K00588	caffeoyl-CoA *O*-methyltransferase	2.1.1.104
				**Stilbenoid, diarylheptanoid, and gingerol biosynthesis**	
*CCoAMT*	Ccrd_012618	KVI08997	K00588	caffeoyl-CoA *O*-methyltransferase	2.1.1.104
				**Carotenoid biosynthesis**	
*NCED*	Ccrd_017614	KVI04081	K09840	9-cis-epoxycarotenoid dioxygenase	1.13.11.51
				**Biosynthesis of various plant secondary metabolites**	
*NAS*	Ccrd_025247	KVH87493	K05953	nicotianamine synthase	2.5.1.43

## Data Availability

Sequencing data are available at NCBI Bioproject n. PRJNA894541.

## Supplementary Materials

The following supporting information can be downloaded at: https://www.mdpi.com/article/10.3390/plants12081600/s1, Figure S1: Comparative fields set-up with sanitized and non-sanitized plants of (a) Locale di Mola tardivo and (b) Troianella. Pictures were taken in early October 2021. In both the pictures, sanitized plants (side rows) show a bigger size and increased plant vigor than non-sanitized counterparts (central row); Figure S2: Varimax rotation of principal component analysis (PCA) score plot of libraries obtained from non-sanitized (NS, blue trace) and sanitized (S, red trace) samples of Locale di Mola (LM) and Troianella (TR) ecotypes. Open circles represent the RNA-seq normalized count values of 26505 genes of *Cynara carduculus*; Figure S3: Chemical structures of the identified phytochemical compounds in high-pressure liquid chromatography with diode array detection (HPLC-DAD) analysis in sanitized (S) and non-sanitized (NS) artichoke samples; Figure S4: High-pressure liquid chromatography with diode array detection (HPLC-DAD) chromatograms of polyphenol extracts obtained from non-sanitized (NS, red trace) and sanitized (S, blue trace) samples of (a) Locale di Mola (LM) and (b) Troianella (TR) ecotypes; Figure S5: Total protein analysis and lignin content calibration line construction. Coomassie brilliant blue staining of native-PAGE to evaluate equal gel loading of (a) soluble (SP) fraction and (b) bound (BP) protein fraction of Locale di Mola (LM) and Troianella (TR) ecotypes. The total amount of proteins loaded (black profile) in non-sanitized (NS) and sanitized (S) samples were analyzed by ImageJ software. (c) Quantification of lignin content was assessed by a calibration line obtained measuring the absorbance at 280 nm from five dilutions (1:2) of 10mg of alkali lignin; Table S1: Quantification of virus titer based on reads coverage on virus genome of three ecotypes of Locale di Mola (LM) and Troianella (TR), non-sanitized (NS) and sanitized (S); Table S2: Gene expression values, FDR and relatives genetic information of Locale di Mola sanitized (LM-S) DEGs compared to non-sanitized (LM-NS) samples; Table S3: Gene expression values, FDR, and relative genetic information of Troianella sanitized (TR-S) DEGs compared to non-sanitized (TR-NS) samples; Table S4: KEGG orthology (KO) description and fragments per million mapped reads per kilobase exon (FPKM) mean values of 103 DEGs in common between Locale di Mola (LM) and Troianella (TR) samples in sanitized (S) vs. non-sanitized (NS) comparison; Table S5: Gene expression values, FDR, and relative genetic information in the comparison between Locale di Mola (LM) and Troianella (TR) non-sanitized (NS) samples (LM-NS vs. TR-NS); Table S6: Gene expression values, FDR, and relative genetic information in the comparison between Locale di Mola (LM) and Troianella (TR) sanitized (S) samples (LM-S vs. TR-S); Table S7: KEGG orthology (KO) description and fragments per million mapped reads per kilobase exon (FPKM) mean values of 56 DEGs in common between non-sanitized (NS) and sanitized (S) samples in Locale di Mola (LM) vs. Troianella (TR) comparison; Table S8: Comparison of differentially expressed *POD* genes between sanitized (S) and non-sanitized (NS) samples of Locale di Mola (LM) and Troianella (TR); Table S9: List of primers used for validation of transcriptome results by qPCR; Table S10: Comparison of differentially expressed gene FC values between sanitized (S) and non-sanitized (NS) samples of Locale di Mola (LM) and Troianella (TR) validated by quantitative real-time PCR (qPCR).
