# Supplementary material for: Comparative Analysis of Bioactive Compounds in Two Globe Artichoke Ecotypes Sanitized and Non-Sanitized from Viral Infections
Source: Plants (Basel). 2023 Apr 10;12(8):1600. doi: 10.3390/plants12081600 (PMC10145195; doi:10.3390/plants12081600)
Supplement: Supplementary file 1 [file plants-12-01600-s001.zip › Supplementary figures.pdf]

Supplementary Figures

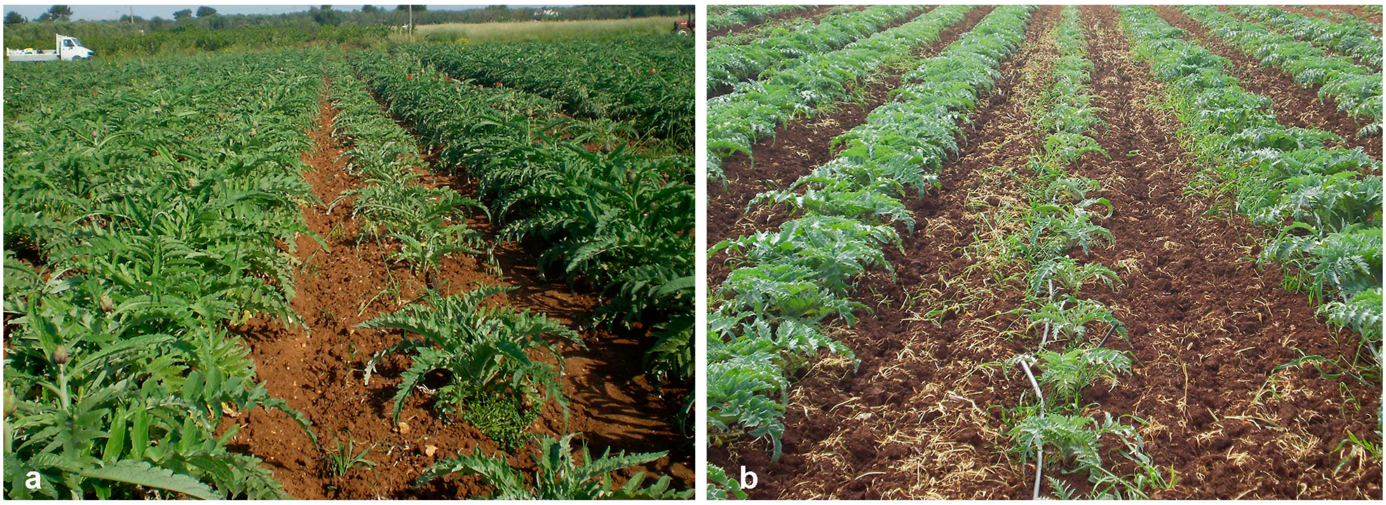

Figure S1. Comparative fields set-up with sanitized and non-sanitized plants of (**a**) Locale di Mola tardivo and (**b**) Troianella. Pictures were taken in early October 2021. In both the pictures, sanitized plants (side rows) show bigger size and increased plant vigor than non-sanitized counterparts (central row).

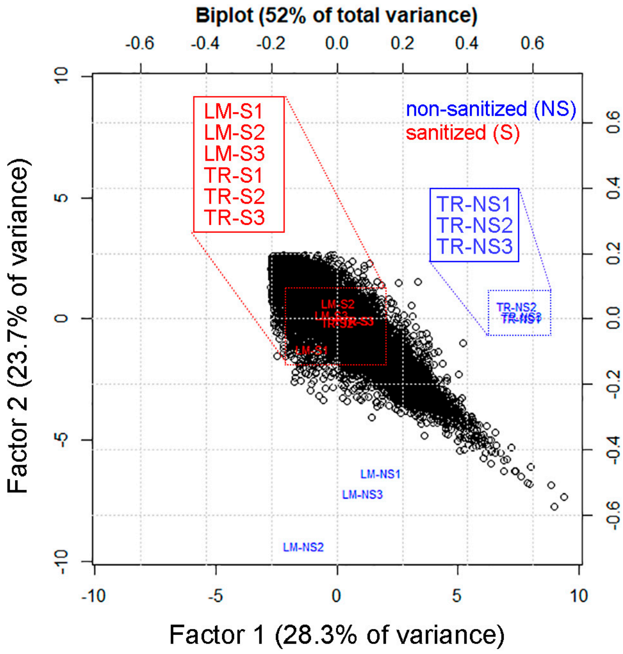

Figure S2. Varimax rotation of principal component analysis (PCA) score plot of libraries obtained from non-sanitized (NS, blue trace) and sanitized (S, red trace) of Locale di Mola (LM) and Troianella (TR) ecotypes. Open circles represent the RNA-seq normalized count values of 26505 genes of *Cynara carduculus*.

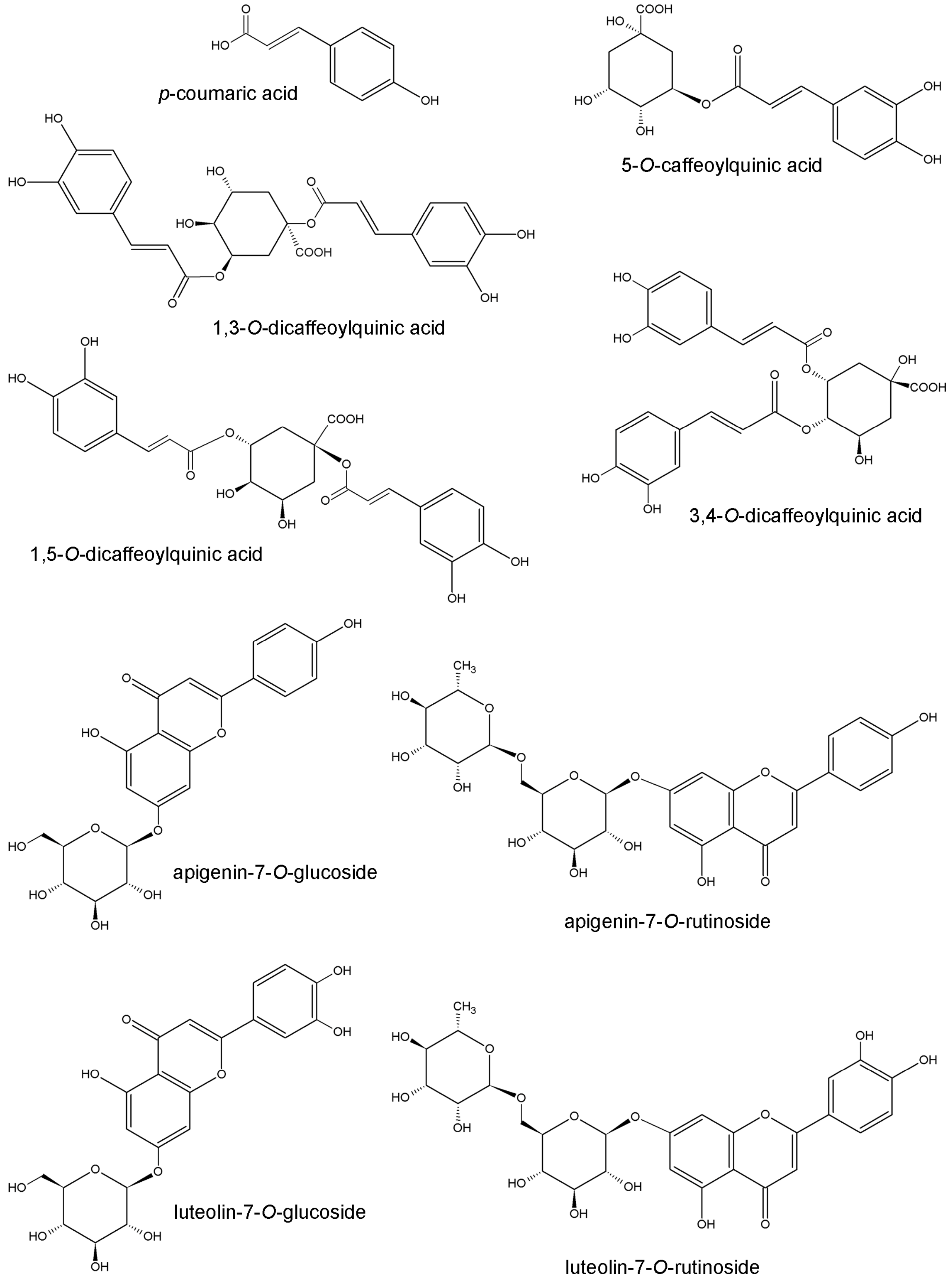

Figure S3. Chemical structures of the identified phytochemical compounds in High Pressure Liquid Chromatography with Diode Array Detection (HPLC-DAD) analysis in sanitized (S) and non-sanitized (NS) artichoke samples.

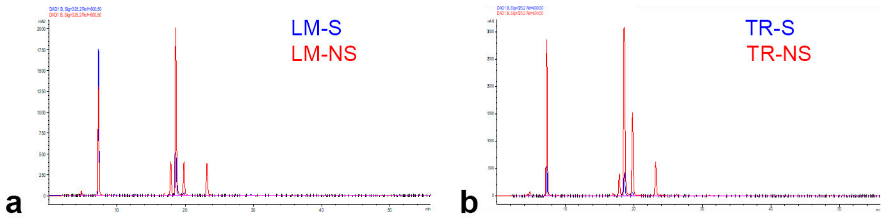

Figure S4. High Pressure Liquid Chromatography with Diode Array Detection (HPLC-DAD) chromatograms of polyphenol extracts obtained from non-sanitized (NS, red trace) and sanitized (S, blue trace) of (**a**) Locale di Mola (LM) and (**b**) Troianella (TR) ecotypes.

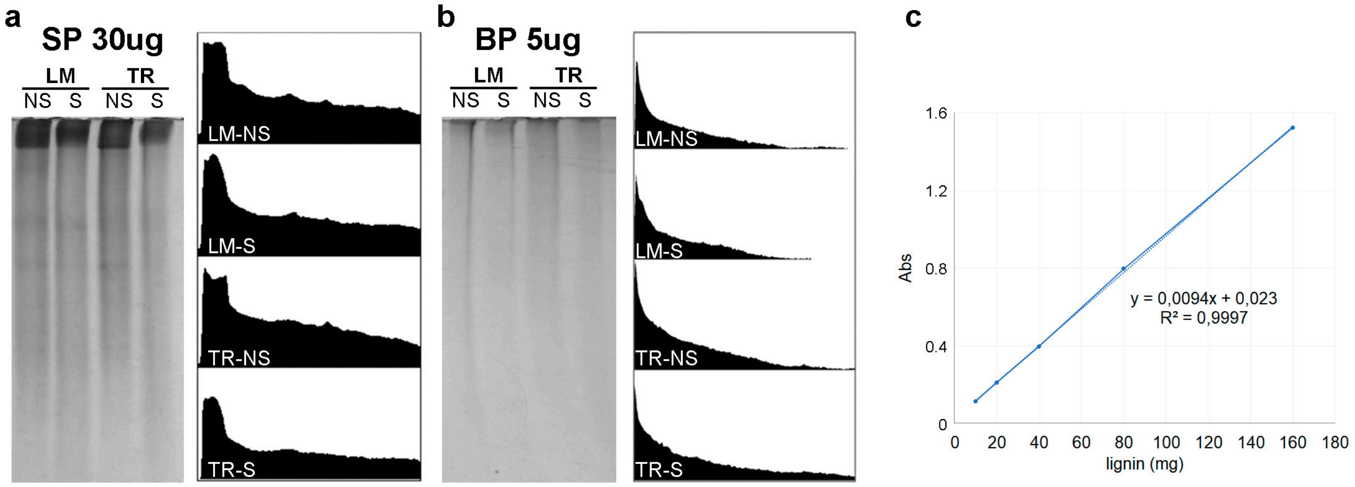

Figure S5. Total protein analysis and lignin content calibration line construction. Coomassie brilliant blue staining of native-PAGE to evaluate equal gel loading of (**a**) soluble (SP) fraction and (**b**) bound (BP) protein fraction of Locale di Mola (LM) and Troianella (TR) ecotypes. Total amount of proteins loaded (black profile) in non-sanitized (NS) and sanitized (S) samples were analyzed by ImageJ software; (**c**) Quantification of lignin content was assessed by calibration line obtained measuring the absorbance at 280 nm from five dilution (1:2) of 10mg of alkali lignin.
